# Islamophobia, mental health and psychiatry: South Asian perspectives

**DOI:** 10.17650/2712-7672-2020-1-1-78-84

**Published:** 2020-09-02

**Authors:** Roy A. Kallivayalil, Abdul Q. Jilani, Adarsh Tripathi

**Affiliations:** Pushpagiri Institute of Medical Sciences; Era’s Lucknow Medical College; King George’s Medical University

**Keywords:** Islamophobia, Muslim Phobia, Muslim Mental Illnesses, Hate Crimes, Psychiatry, Discrimination, Mental Health, South Asia, исламофобия, мусульманофобия, психические заболевания у мусульман, преступления на почве нетерпимости, психиатрия, дискриминация, психическое здоровье, Южная Азия

## Abstract

Asia is the largest and the most populous continent on earth. South Asia has a population of around 1.8 billion,thus constituting about one fourth of humanity. India, Pakistan, Bangladesh, Sri Lanka, Nepal, Bhutan, the Maldives and Afghanistan are the countries in South Asia and many of them are Muslim-majority nations. Although India is predominantly a Hindu nation with a total population of 1.4 billion, there are more Muslims in India than in Pakistan and other South Asian nations. Hindus, Muslims, Christians, Sikhs and followers of other religions have lived peace fully in South Asia for centuries. However, certain incidents of communal violence and other untoward occurrences in SouthAsia suggest that Islamophobia is present here too. The authors discuss demography, cultures and the possible effect of Islamophobia on the mental health of the people of South Asia.

## INTRODUCTION

“Usually the term phobia refers to the psychological fear of the human mind from something that poses a threat. But when a species starts using the term fear against a biological portion of itself, there is nothing more demeaning than this."— Abhijit Naskar (The Islamophobic Civilization)

The origin of Islam as a religion/civilization in the year 610 CE in Saudi Arabia, added a novel religious faith and culture to the many existing contemporary religions [1]. Over a period of time, Islam attracted people across the boundaries of nations, geographical locations, cultures and creeds, and has spread rapidly in the past 1,400 years emerging as the second largest religious group in the world, today constituting 24% of the world population [2].

In contrast, in the past few decades there has been a rise in anti-Muslim sentiment and global hatred [3]. The basic principle of Islam remains unchanged since its inception and there could be several reasons for the rising anti-Muslim sentiment. One reason could be the difference between the basic principles of Islam and the way it is followed. There is a strong possibility that, due to a lack of religious knowledge, poverty and ignorance, the followers of Islam may have distorted Islamic rituals and culture [4–6]. The repercussions of inflexible attitudes and cultures of various religions may be responsible for rising anti-Muslim sentiment. The other reason could be that others misunderstand Islam's principles, practices, cultures and rituals. In addition, there might be various reasons such as cross-border terrorism, economic exclusion, war for sovereignty and materialistic gains, along with maintenance of supremacy between countries of the region, e.g., between India and Pakistan, giving religious colours to these conflicts [7].

Fortunately, despite wide variation in religious beliefs, with the cohesiveness and respect of cultural differences and religious views, the South Asian society has been considered resistant to religious hatred for centuries [8], but this bond seems to be weakening. The recent increase in religious intolerance in South Asia is a matter of serious concern.

South Asia is already facing various challenges in raising the standard of living of its people and in terms of economic growth, social progress and cultural development [9] . Alongside these existing challenges, rising Islamophobia deserves special attention as a major threat to health outcomes and health disparities in South Asia with the largest population of Muslims in the world [10]. There is a need for intervention with social psychiatry initiatives to prevent rising Islamophobia and religious intolerance which acts as a persistent chronic stressor for the whole community. This will also prevent the emergence of mental health problems due to Islamophobia.

## DEMOGRAPHY OF SOUTH ASIA AND THE MUSLIM POPULATION

Eight countries, Afghanistan, Bangladesh, Bhutan, the Maldives, Nepal, India, Pakistan, and Sri Lanka, together form South Asia and are known as the South Asian Association for Regional Cooperation (SAARC) [11]. This area constitutes 3.4% of the world's land surface area with one fourth (1.8 billion) of the world's population. Thus, it is the most densely populated geographical region in the world with a significant proportion of Muslims [9, 12].

South Asia has many major religions such as Hinduism, Islam, Christianity, Jainism, Buddhism and Sikhism. About 63% (about one billion) of the population of South Asia are Hindus, 31% (600 million) are Muslims and the rest are Buddhists, Jains, Christians and Sikhs [13, 14]. The Hindus, Buddhists, Jains, Sikhs and Christians are concentrated in India, Nepal, Sri Lanka and Bhutan, whilst the Muslims are concentrated in Afghanistan (99%), Bangladesh (90%), Pakistan (96%) and the Maldives (100%) [14]. Among countries with a dominant non- Muslim population, Muslims constitute 14.5% of India, 12.61% of Sri Lanka and 4.4% of Nepal. It is to be noted that, in Muslim dominated countries, Hindus constitute 2% of the population of Pakistan, 9% of the population of Bangladesh and less than 1% of the population of Afghanistan [9]. This religious demography of South Asia could be one of the reasons for religious discrimination in this region. Although there is a dearth of official/scientific data, as per reports in electronic/ print media, Islamophobia is presumed to be present here as well.

## ISLAMOPHOBIA: CONCEPT, CAUSES AND PREVALENCE

### Concept of Islamophobia

The concept of Islamophobia emerged from Western nations [3, 15], where Muslims are a minority.

Unfortunately, it is spreading rapidly to encompass the whole of the world including Muslim dominated Asian regions. Furthermore, despite the huge Muslim population, there is a dearth of scientific research and official data from developing and Asian countries regarding the consequences of rising Islamophobia for Muslims and other communities [3] .

Islamophobia is defined as an intense dislike or fear of Islam and its followers, especially as a political force; hostility or prejudice towards Muslims [16]. The term Islamophobia emerged in the 1970s and later gained widespread currency. It reached public policy prominence in Western countries after the 1997 report by the Runnymede Trust Commission on British Muslims and Islamophobia entitled "Islamophobia: A Challenge For Us All" [15].

The introduction of the term was justified by the report's assessment that "anti-Muslim prejudice has grown so considerably and so rapidly in recent years that a new item in the vocabulary is needed" [17]. In recent times, Islamophobia has been conceptualized as social stigmatization of Islam and Muslims, dislike of Muslims as a political force and a distinct construct referring to anti-Muslim stereotypes, racism, or xenophobia [18, 19]. Although, anti-Muslim sentiment is increasingly common globally, it has taken a form of social stigma in the Western world where Islamophobic sentiment has already gained scientific attention, particularly after the terrorist attacks of 11. September, 2001 [3].

### Causes and Prevalence of Islamophobia

The term Islamophobia gained prominence following terrorist attacks such as 9/11 in the United States, the Taliban's fundamentalist proscriptions and restrictions in Afghanistan, the Charlie Hebdo attack in France and the emergence of the self-proclaimed Islamic State group (ISIS) which allegedly showed videos of the beheading of their prisoners who were often journalists [20, 21].

The concept of Islamophobia became a global theme. With the advancement of communication/information technology and the role played by the media, Muslims were often described as fanatics, irrational, primitive, belligerent and dangerous for modern society and other religions [22]. There was negative portrayal of Muslims in many countries including those in Asia, which influenced the attitudes of common people [23].

In South Asia, the series of various heinous terrorist attacks, especially in India, e.g., 2000 Red Fort attack, 2001 Indian Parliament attack, 2008 Mumbai attack, 2008 bombing series in Delhi and 2016 Uri attack in Kashmir, mostly sponsored by cross-border terrorist organizations, fuelled the concept of Islamophobia [24–26]. India is the largest and fastest growing economy in this region with an 82% share of the South Asian economy. It is the only member of the powerful G-20 major economies from the region. Any terrorist activity against India is widely covered, it negatively affects the whole of South Asia [9] and it contributes to the rising tide of Islamophobia. Similarly, the 2002 beheading in Pakistan of the American journalist Daniel Pearl, the 2002 Karachi bus bombing carrying French engineers, the 2003 and 2006 attacks targeting the U.S. consulate in Karachi, the 2016 Lahore suicide bombing targeting Christians and several other such incidents where extremist Muslim organizations were primarily responsible, further expanded the concept of Islamophobia [27]. Some of the attacks in Pakistan and Afghanistan are believed to be attacks against other sub-groups within the Muslim community itself. The 2011 killing of Osama bin Laden, the head of the Islamist group Al-Qaeda, may also have contributed to the rise of Islamophobia in the West [28].

The religious demography of South Asia is important from the point of view of rising Islamophobia [29]. In some of the populous countries in the region, nationalism is sometimes judged by the hostility towards the neighbouring country and its predominant religion [30, 31]. In this way, the minority populations of both the countries, i.e., Hindus in Pakistan and Muslims in India, are at greater risk of discrimination by the majority. This could be one of the major reasons for the alleged rising Islamophobia and hate crimes against Muslims in India, and similarly, the rising hate crimes against Hindus in Pakistan.

At present, the concept of Islamophobia seems to be ingrained in the lifestyle of Western societies [32]. A post-9/11 poll in the United States reported that 60% of Americans had unfavourable attitudes toward Muslims. Many Americans relate to Muslims with fear- related terms such as violence, fanatic, radical, war and terrorism [33]. While Muslim immigration may be the cause for Islamophobia in the West, the large number of minority Muslims in some countries in South Asia could be the cause in this region. [34]

Although the common perception is that Islamophobia is growing in South Asia too, there is a lack of scientific evidence to support this argument [23]. Many believe that there has been a growing feeling of unease and insecurity amongst Muslims in India [35]. A recently published meta-analysis [23] examining the role of the media in the construction of a Muslim and Islamic identity, showed that Muslims are generally negatively framed and their civilization, i.e., Islamic faith, is predominantly portrayed as a violent civilization/religion. Similar views have been put forward by China about the media portraying negative stereotypes of Muslims [36]. In the present era of technology, where any true/false information can reach across the globe within a fraction of a second, social media platforms have been a viable medium to spread hatred among communities. There are thousands of groups/web pages by people with a similar mindset/religion who share and spread negatively framed news against people of other faiths [37, 38]. It is well known that when one set of people of a particular faith/group post provocative or inaccurate news on social media, others will reciprocate in a spirit of revenge, with the consequence of an exponential increase in hatred. Negative media campaigns have the potential to generalize Islamophobic attitudes towards the global Muslim community. This leads to feelings of insecurity among minority Muslims, and a distorted self-perception as non-peace loving people. To add to this, recent terrorist activities are usually linked with extremist elements of the Muslim communities, both globally and in South Asia [27]. Consequently, there can be reciprocal negative psychological responses against common Muslims by the majority, based on widespread news/media coverage of such terrorist activities.

## ISLAMOPHOBIA AND MENTAL HEALTH PROBLEMS

The recent rise in terrorism has led to widespread fear of Islamophobia among followers of Islam and other religions. The constant fear, agony, hatred, apprehension, etc., will definitely have a psychological impact on all people, as human beings are socially inseparable.

Islamophobia can negatively influence health by disrupting many systems, i.e., individual (stress reactivity and identity concealment), interpersonal (social relationships and socialization processes) and structural (institutional policies and media coverage) [3] . However, there is a dearth of scientific data on this topic from South Asian countries. Like many traumatic events, e.g., family disputes, interpersonal conflicts, death of loved ones, earthquake, major road traffic accidents, chronic physical diseases and wars, the impact of Islamophobia will also be traumatic on the concerned individuals and groups.

Islamophobia can also be a source of stress. Although, the seeds of psychological problems may be implanted well before birth in the form of genetic predisposition, significant environmental stressors, social support and coping skills play important roles in the causation/ precipitation of the majority of mental health problems [39, 40]. Stress has been reported to play a major aetiological role in acute stress reaction and adjustment disorders, and a precipitating role in schizophrenic episodes [41]. Hence, it could be inferred that a variety of mental disorders are associated with significant stress [39, 42, 43]. Islamophobia acts as a significant source of stress as it encompasses a range of anti- Muslim sentiments varying from derogatory remarks, discrimination and stigmatization to hate and targeted crimes against Muslims [44].

Stressors have a major influence on mood, sense of wellbeing, behaviour and health [45]. From a psychological point of view, psychological defence mechanisms may have significant adverse effects among Muslims regarding Islamophobia. Introjection occurs when a person internalizes the beliefs of other people, i.e., Muslims would start to see themselves in the negative way that they are portrayed by the media. On the other hand, the psychological defence mechanisms which may play a role against Muslims among the general population are rationalization, symbolization and reaction formations. The majority may develop the belief that since most of the terrorist attacks across the world have somehow been linked with Muslim organizations, Muslims can be considered as a symbol of terrorism and thus hate crimes against Muslims could, to a certain extent, be justified. Hate crimes like mob lynching may be a significant risk factor for stress-related disorders in people from a concerned community, e.g., acute stress reactions, grief reactions, insomnia and adjustment disorders among close family members, post-traumatic stress disorders among survivors or witnesses [46, 47].

In physical disorders, the majority of laboratory investigations are carried out to look for the causes/aetiologies of various disorders. In psychiatry, a detailed history and assessment focuses on delineation of specific stress or perpetuating factors. There are many scales to measures various types of significant stress in psychiatric patients such as the Perceived Stress Scale [48] and the Holmes-Rahe Stress Inventory [49]. These scales measure the types and severity of stress. These scales also explore the degree to which situations in one's life are appraised as stressful. In this regard, scales have also been developed to measure Islamophobia regarding fearful attitudes towards, and avoidance of Muslims and Islam, arguing that Islamophobia should "essentially be understood as an affective part of social stigma towards Islam and Muslims, namely fear" [50, 51].

Mental disorders or psychological stress are associated with various physical disorders like hypertension, diabetes mellitus, obesity, peptic ulcer disease, etc. [52–55]. The Centre for Disease Control and Prevention in the United States has estimated that stress accounts for about 75% of all visits to the doctor [56]. In Islamophobia, there are no adequate resources to cope with the stressor, leading to the perception of being under stress [64]. Thus, there may be serious health problems due to rising Islamophobia in South Asia.

Furthermore, this rising intolerance to religious cultures/beliefs around the world may have an adverse effect on the personality of growing children and adolescents [57]. The growing phase of childhood and adolescence is itself a period of stress, in which children are dealing with the challenges of going through puberty, meeting changing expectations, school performance and coping with new feelings. Most children meet these challenges successfully and grow into healthy adults while others may have a harder time coping with their problems. The rising incidence of hate speeches/crimes along with feelings of discrimination and stigmatization will have a negative impact on mental health. Exposure to stress during childhood and adolescence has lasting neurobiological effects with psychological consequences, e.g., deregulation of affect, anxiety and mood disorders, provocative and aggressive behaviours, the avoidance of intimacy, disturbances in attachment, post-traumatic stress disorder and depressive symptoms [58–60].

Islamophobia can affect other aspects of life too. However, there is no scientific research that can reveal the impact of Islamophobia in terms of discrimination in the health service, workplace, job opportunities and in terms of the increased scrutiny in South Asian countries on the basis of appearance and religious background [61, 62]. Literature from Western countries regarding how Islamophobia affects the mental health of Muslims [20, 63] indicates that religious prejudice in the form of Islamophobia is a major obstacle to Muslims' integration because it increases the incongruity between majority and minority members' acculturation attitudes. In the West, various forms of religious stigma can affect Muslims' national identity and engagement in the public and private sphere in distinct ways. In the absence of scientific data, we are unable to draw such conclusions for South Asia.

Communal and religious harmony is important for the social, cultural and economic growth of countries. It is also imperative to protect the human rights of all and especially of minorities.

## CONCLUSION

South Asia has a huge and diverse population and Islamophobia is a matter which needs attention and further study in this region. The media often portrays Muslims negatively. Islamophobia is associated with significant stress and anxiety and the prevalence of mental health problems could be expected to increase, affecting Muslims and other communities. Islamophobia is also likely to colour the phenomenology of mental disorders.

Mental health is of low priority and its role on human and societal wellbeing is underestimated in most developing countries. Many South Asian countries are already fighting joblessness, poverty, illiteracy, cultural myths, superstition and inadequacy of medical facilities. The rise of Islamophobia will add another major challenge and there is a need to study the perceptions of an Islamophobic society, experiences of religious discrimination and negative representations of Muslims. The recent rise in hate crimes against Muslims in South Asia calls for a public health perspective that considers the stigmatized identity of Muslims and the health implications of Islamophobic discrimination.

Islamophobia can be a double-edged sword, with demoralizing effects on the psychology of human beings irrespective of their religious faith. The more it rises, the more severe will be the after-effects and there will be an increased likelihood of psychological illness and violation of human rights. The prompt awareness and tackling of rising Islamophobia will promote social, economic and personal growth of the people of South Asia.

## Conflict of Interest

Authors declare no conflict of interest.
